# Chromosome-Level Genome Assembly and Annotation of a Periodical Cicada Species: *Magicicada septendecula*

**DOI:** 10.1093/gbe/evae001

**Published:** 2024-01-08

**Authors:** Jonas Bush, Cynthia Webster, Jill Wegrzyn, Chris Simon, Edward Wilcox, Ruqayya Khan, David Weisz, Olga Dudchenko, Erez Lieberman Aiden, Paul Frandsen

**Affiliations:** Huck Life Sciences Institute, The Pennsylvania State University, State College, PA, USA; Department of Plant and Wildlife Sciences, Brigham Young University, Provo, UT, USA; Department of Ecology and Evolutionary Biology, University of Connecticut, Storrs, CT, USA; Department of Ecology and Evolutionary Biology, University of Connecticut, Storrs, CT, USA; Department of Ecology and Evolutionary Biology, University of Connecticut, Storrs, CT, USA; Department of Plant and Wildlife Sciences, Brigham Young University, Provo, UT, USA; The Center for Genome Architecture, Department of Molecular and Human Genetics, Baylor College of Medicine, Houston, TX, USA; The Center for Genome Architecture, Department of Molecular and Human Genetics, Baylor College of Medicine, Houston, TX, USA; The Center for Genome Architecture, Department of Molecular and Human Genetics, Baylor College of Medicine, Houston, TX, USA; The Center for Theoretical Biological Physics, Rice University, Houston, TX, USA; The Center for Genome Architecture, Department of Molecular and Human Genetics, Baylor College of Medicine, Houston, TX, USA; The Center for Theoretical Biological Physics, Rice University, Houston, TX, USA; Broad Institute of MIT and Harvard, Cambridge, MA, USA; Department of Plant and Wildlife Sciences, Brigham Young University, Provo, UT, USA; Data Science Lab, Office of the Chief Information Officer, Smithsonian Institution, Washington, DC, USA

**Keywords:** cicada, genomics, assembly, annotation, Hemiptera, HiC

## Abstract

We present a high-quality assembly and annotation of the periodical cicada species, *Magicicada septendecula* (Hemiptera: Auchenorrhyncha: Cicadidae). Periodical cicadas have a significant ecological impact, serving as a food source for many mammals, reptiles, and birds. *Magicicada* are well known for their massive emergences of 1 to 3 species that appear in different locations in the eastern United States nearly every year. These year classes (“broods”) emerge dependably every 13 or 17 yr in a given location. Recently, it has become clear that 4-yr early or late emergences of a sizeable portion of a population are an important part of the history of brood formation; however, the biological mechanisms by which they track the passage of time remain a mystery. Using PacBio HiFi reads in conjunction with Hi-C proximity ligation data, we have assembled and annotated the first whole genome for a periodical cicada, an important resource for future phylogenetic and comparative genomic analysis. This also represents the first quality genome assembly and annotation for the Hemipteran superfamily Cicadoidea. With a scaffold N50 of 518.9 Mb and a complete BUSCO score of 96.7%, we are confident that this assembly will serve as a vital resource toward uncovering the genomic basis of periodical cicadas’ long, synchronized life cycles and will provide a robust framework for further investigations into these insects.

**Significance**

Periodical cicadas have an outsized cultural and ecological impact and a highly unusual long, synchronized, periodical life cycle that holds many yet-to-be-resolved scientific mysteries. The assembly we report here is the first whole genome *Magicicada* assembly, the first complete genome assembled from a species in the plant-sucking-bug family Cicadidae (∼3,000 described species), and the first whole genome assembled for the Hemipteran superfamily Cicadoidea, which dates back ∼250 Mya.

## Introduction

Periodical cicadas are plant-sucking bugs in the order Hemiptera—the genus *Magicicada* contains 7 species, which are distributed widely across the eastern United States. They range from Nebraska to Texas at the eastern edge of the Great Plains, to the Atlantic coast from Massachusetts to Georgia (Simon 1988). The genus can be divided into 3 morphologically distinct species groups: Decim, Decula, and Cassini. These 3 species groups each contain one 17-yr species and one or two 13-yr species. During their nymphal stages, cicadas feed underground on the xylem fluid of tree roots, and after 13 or 17 yr, emerge in large year classes called “broods,” which are composed of multiple different *Magicicada* species (Williams and Simon 1995; Simon et al. 2022). These emergences have a major ecological impact—for example, many avian species rely heavily on adult cicadas to feed their young and even experience population growth corresponding with a brood emergence (Koenig and Leibhold 2005; Pons 2020). Periodical cicadas are one of the quintessential examples of the evolutionary strategy of predator satiation, which is likely one of the selective pressures that influenced the development of such unique life cycles (Karban 1982; Williams et al. 1993; Koenig and Leibhold 2013). *Magicicada septendecula* is one of three species belonging to the Great Eastern Brood, or Brood X, which last emerged in 2021 and will reappear in the next generation in 2038 (Kritsky 2021).

Several mysteries surround the evolution of these fascinating insects, including the origins of broods, the mechanisms by which they can track the passage of time, the well-documented large emergences of a portion of a population exactly 4 yr early or late and their incredibly lengthy life cycles. For most of these questions, in-depth investigations have been hindered by the lack of quality genomic resources (Berlocher 2013; White and Pirro 2021; Simon et al. 2022). Although separated into multiple different broods, the members of each species group have experienced gene flow, presumably during coemergences made more frequent by the 4-yr early and late individuals (Fujisawa et al. 2018). Despite this gene flow, life cycles have maintained their integrity. Previous studies have used mRNA sequencing to investigate gene flow and construct molecular phylogenies and have found that between 13- and 17-year species pairs, genomic divergence is minimal except in the Decim group where one of the species, *Magicicada tredecim*, is reproductively isolated from the other two, *Magicicada septendecim* and *Magicicada neotredecim* (Fujisawa et al 2018).

Little is known about the demographic history and mechanism(s) of speciation within *Magicicada*. Several hypotheses have been proposed to explain multiple speciation events that have occurred in the history of these insects, most notably population fragmentation due to glacial events (Fujisawa et al 2018; Sota et al. 2013). Because periodical cicadas have been shown to time their emergence by measuring cumulative soil temperature (Heath 1968), glacial events in the last million years could provide a convenient explanation and impetus for speciation events by inducing early or late emergences that could lead to reproductively isolated populations (Cox and Carlton 1988). Scattered early and late bloomers, so to speak, have been observed appearing 1 or 2 yr before or after broods and large proportions of populations have been observed to emerge 4 yr early or late (Cooley et al. 2018; Simon et al. 2022).

While there is strong evidence that cicadas time their crawl to the surface based on accumulated soil temperature (Heath 1968), there are only unsupported hypotheses to explain how they can track the passage of years. One of the most promising hypotheses involves biological pathways that are triggered by changes in the chemical makeup of xylem fluid as the seasons change (Lloyd and Dybas 1966; Heath 1968). Annotated genome assemblies for *Magicicada* species will allow the discovery of genes that may be involved in these hypothesized pathways and could also allow for the comparison of genome sequences with other periodical species (Helioväära et al. 1994) or early/late bloomers to find gene variants that could explain the divergence in behavior. As this is one of nature's most sophisticated biological clocks, the results will be fascinating and provide insights into the mechanisms whereby other species track the passage of time. Recent research into the genetic control of periodicity in long-lived bamboo (Zhang et al. 2021) and temperature-based RNA expression and editing (Birk et al. 2023) could also provide fascinating hypotheses of the molecular mechanisms for timekeeping in this genus.

**Fig. 1.**
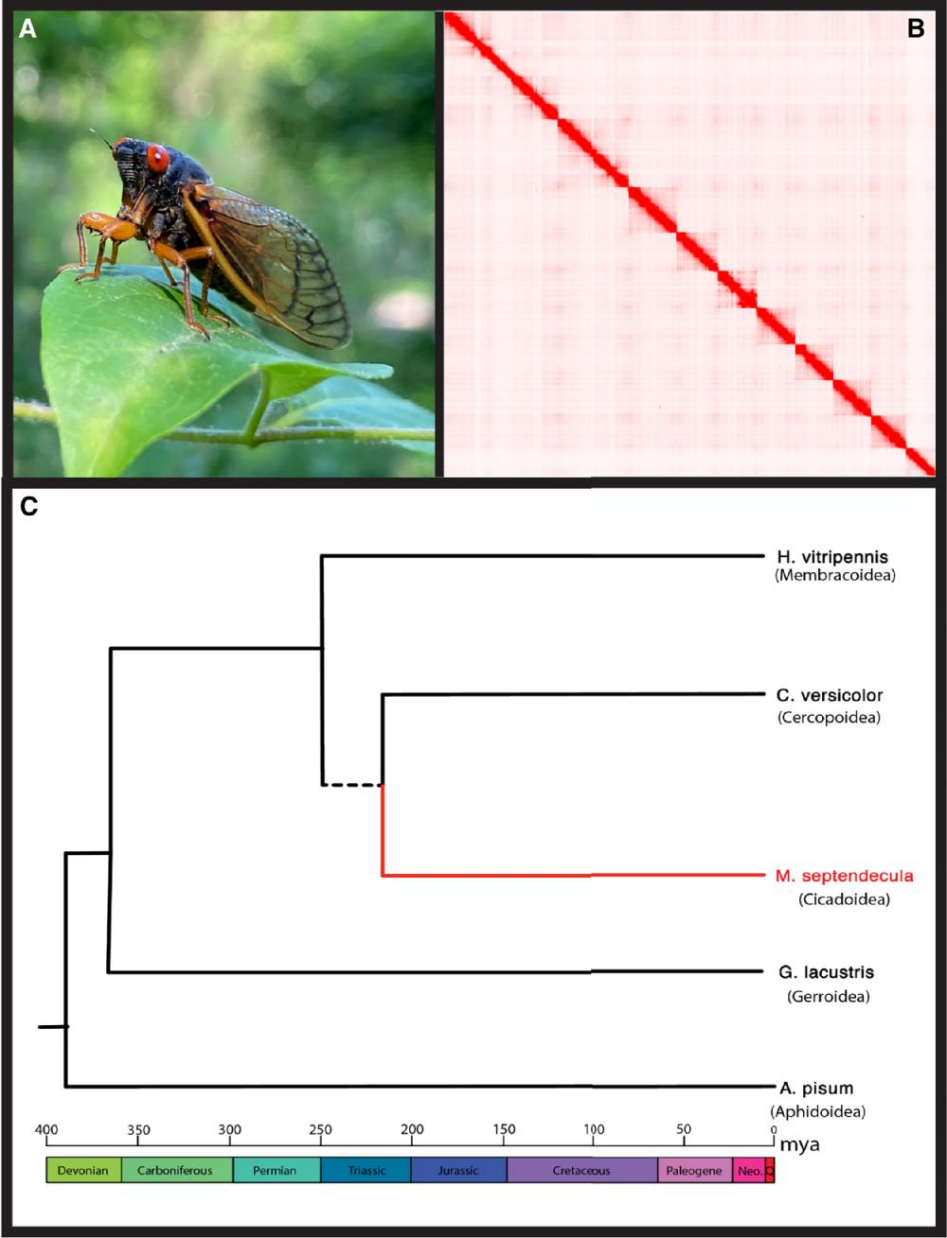
A) Photograph of *M. septendecula*, Herndon, VA (Photo credit: Paul Frandsen). B) Contact map generated from Hi-C data showing 10 distinct blocks corresponding to chromosomes (2*n* = 19/20). The link for the full interactive figure can be found in the Supplementary Material (see Table S6). C) Phylogenetic tree illustrating the relationships between the included Hemipteran superfamilies (see Table 1) with relative branch lengths illustrating divergence dates estimated by Johnson et al. (2018). The dotted line subtending Cicadoidea and Cercopoidea indicates uncertainty in superfamily relationships as discussed by Skinner et al. (2020), Cao and Dietrich (2022), and Song and Zhang (2023).

Further highlighting the importance of this genomic resource is the ancient estimated divergence time of the superfamilies Cercopoidea and Cicadoidea (Johnson et al. 2018). This divergence represents 250 million years of evolution, which are unrepresented (Fig. 1C) in the current genomic data. Here, we fill this gap by sequencing, assembling, and annotating a chromosome-length genome for the periodical cicada, *M. septendecula*, commonly known as the “little 17-year cicada.”

## Results and Discussion

### HiFi Assembly and Scaffolding with Hi-C Data

We sequenced a single individual male from the periodical cicada species, *M. septendecula*. Upon assembly, we found that the genome length is quite large compared to most other insects as measured by flow cytometry data (Hanrahan and Johnston 2011). At 6,521,820,903 bp, this genome assembly is almost 2.5× the size of the next largest Hemipteran assembly currently on NCBI (Biello et al. 2020). Repetitive elements were found to make up a large percentage of the genome, with a repeat content of 72.78% (35.64% classified) and a GC content of 35.25% as identified by RepeatMasker (v.4.1.2) (Flynn et al. 2020). Analysis with BlobTools (v.1.1.1) (Laetsch and Blaxter 2017) revealed several sequences that were categorized into Chordata as well as viral sequences (see supplementary fig. S1, Supplementary Material online)—however, due to their considerable length and a match in GC content with the rest of the genome, we argue that it is likely that these sequences were misclassified due to a lack of database coverage, similar to findings in the genome assembly of the dragonfly *Tanypteryx hageni* (Tolman et al. 2023).

Hi-C sequencing revealed 9 autosomes with 1 X chromosome (Fig. 1B; supplementary table S1, Supplementary Material online) in an XX/X0 sex-determining system, consistent with prior karyotyping of the genus *Magicicada* (Karagyan et al. 2020). PacBio HiFi sequencing and assembly followed by Hi-C scaffolding resulted in a highly improved genome assembly, with a scaffold N50 of 518.9 Mb, an L50 of 4, and 2,030 total scaffolds. Hi-C scaffolding thus improved our initial assembly into a strong genomic resource as potential pitfalls due to high repeat content (73%) and size (6.5 Gb) are addressed by the long-read sequencing technology as well as scaffolding with Hi-C data. Using NCBI's FCS tool, we trimmed and removed any contigs flagged as contaminants, ending with 10 chromosome-length scaffolds (95.49% of the assembly length) and 2,005 unplaced scaffolds (4.51% of the assembly length). We used tidk (v.0.2.41) (Brown et al. 2023) to search for telomeric repeats in the assembly and found telomeres on the ends of each chromosome-length scaffold (see supplementary fig. S2, Supplementary Material online). The complete BUSCO score for the Hi-C-scaffolded assembly was 96.7%.

### Annotation

Five Illumina, paired-end *Magicicada* RNA-Seq libraries were trimmed and aligned to the *M. septendecula* genome (supplementary table S2, Supplementary Material online). Prior to quality control, total reads ranged between 22,101,494 and 85,009,400. Following quality control, that range fell between 22,092,126 and 85,000,848. RNA alignment rates were variable, falling between 81.29% and 94.83%.

The EASEL pipeline, which leverages generalized hidden Markov models and random forests to both predict and refine gene models, produced an unfiltered and filtered structural annotation. The unfiltered prediction, derived from multiple levels of RNA and protein support, captured 140,729 genes and 303,929 transcripts. This duplication was intentionally high to maximize gene sensitivity for downstream filtering. The mono:multiexonic ratio of 2.07 was indicative of fragmentation; however, despite an inflated number of false positives, 99.7% (S: 20.8%, D: 78.9%) of Insecta single-copy orthologs were captured. Following primary and secondary feature filtering using the invertebrate training set, EASEL predicted 22,785 genes and 83,621 transcripts with a mono:multiexonic ratio of 0.200 and a BUSCO completeness score of 96.4% (S: 24.9%, D: 71.5%). The functional annotation generated by EnTAP produced 57,260 unique RefSeq similarity search alignments (68.5%). With the addition of EggNOG gene family assignment, 81,978 sequences out of 83,621 were uniquely annotated (98.0%) (supplementary table S3, Supplementary Material online). The primary gene model (longest isoform) resolved the BUSCO duplication rate for the annotation but at the expense of completeness dropping to 91.2% (S: 89.2%, D: 2.0%), the mono:multiexonic ratio increasing to 0.209, and RefSeq alignments dropping to 59.4% (supplementary table S4, Supplementary Material online). This is slightly lower than the assembly BUSCO reported previously but still within a range that indicates a high-quality annotation.

## Materials and Methods

### Specimen Collection

Samples were collected and flash frozen on dry ice by Chris Simon and Stephen Chiswell in Knox County, TN and Wilkes Co., NC in May of 2021, during the Brood X emergence. After sequencing, the samples were stored as vouchers in the Bean Life Science Museum at Brigham Young University. For the complete sample metadata, please see Table S5.

### Library Prep and Sequencing

We extracted DNA from a single Wilkes Co., NC male using the Qiagen GenomicTip high molecular weight DNA extraction kit. We then sheared the DNA to 18-kb fragments using a Diagenode Megaruptor and size-selected fragments of >10 kb using a SAGE Science BluePippin. We then generated a HiFi sequencing library using the PacBio SMRTbell Express Template Prep Kito 2.0. We sequenced the library across four 30-h SMRT cells on the PacBio Sequel II instrument at the BYU DNA sequencing center. Hi-C libraries were prepared and sequenced on an Illumina NextSeq by DNAZoo at Baylor College of Medicine using methods described in earlier publications (Rao et al. 2014; Dudchenko et al. 2017; Lamb et al. 2021; Tolman et al. 2023). A Knox Co., TN male was used for Hi-C library preparation.

### Assembly Generation and Contamination Screening

We used PacBio SMRTtools to generate HiFi reads from the raw PacBio subreads, which were then assembled into contigs using hifiasm (v.0.16.1) (Cheng et al. 2021). Raw PacBio reads were aligned and scaffolded to Hi-C reads using Juicer and 3D-DNA, respectively (Durand et al. 2016; Dudchenko et al. 2017). Contact maps were manually inspected using Juicebox Assembly Tools (Dudchenko et al. 2018). We used BLAST (v.2.12.0+) (Camacho et al. 2009) to create a sequence database from the initial assembly and RepeatModeler (v.2.0.1) and RepeatMasker (v.4.1.2) (Flynn et al. 2020) to identify and classify repetitive elements. We used BUSCO (v.5.2.2) (Manni et al. 2021) to evaluate gene completeness in the initial assembly and in the final annotated genome. The genome was checked for contamination using NCBI's FCS tool (v.0.4.0) (Astashyn et al. 2023), and contigs flagged as potential contaminants were removed from the final assembly. Additional sequences marked as contamination were trimmed using the faidx tool in SAMtools (v.1.15.1) (Danecek et al 2021).

### Structural and Functional Annotation

Five public Illumina HiSeq RNA-Seq libraries (paired-end) of the genus *Magicicada* were accessed from NCBI (Table 1). Each library was trimmed with FastP (v.0.23.2) and aligned to the soft-masked *M. septendecula* genome with HISAT2 (v.2.2.1) (Chen et al. 2018; Kim et al. 2019). To generate the structural annotation, the soft-masked genome, RNA-Seq data and Hemiptera OrthoDB (v.11) proteins were provided to EASEL (v.1.4) (Kuznetsov et al. 2023; Webster et al. 2023). The EASEL pipeline assembled transcriptomes via StringTie2 (v.2.2.1) and PsiCLASS (v.1.0.3) and isolated complete open-reading frames with TransDecoder (v.5.7.0), culminating in the generation of a gene model (Haas 2016; Kovaka et al. 2019; Song et al. 2019). Putative transcripts and proteins were aligned to the genome with GMAP (v.2021.08.25) and miniprot (v.0.11), respectively, and converted into hints (Wu and Watanabe 2005; Li 2023). With the provided gene models and hints, AUGUSTUS (v.3.5.0) was run, resulting in an unfiltered structural annotation with alternative transcripts (Stanke et al. 2006). These transcripts were classified by primary and secondary features and filtered via a random forest algorithm using the invertebrate training set and a regressor threshold of 65. Gene-prediction accuracy of filtered and unfiltered models was summarized by the total number of genes and transcripts output by AGAT (v.1.0.0), the mono:multiexonic ratio of genes, BUSCO completeness (insecta_v10) (v.5.4.4), and a 70/70 reciprocal BLAST functional annotation rate output by EnTAP (1.0.1), referencing the complete RefSeq database (v.208) (Hart et al. 2020; Dainat et al. 2022). The final structural and functional annotations were derived from the filtered EASEL output; however, to assess BUSCO duplication rates without the added noise of alternative transcripts, the longest isoform was also extracted.

**Table 1:** Hemipteran genome assembly statistics

Order	Superfamily	Species	Source	GenBank Accession	Assembly Length (Mb)	N50 (Mb)	Assembly BUSCO Results (OrthoDB: Insecta)	Genes Annotated
Hemiptera	Cicadoidea	*Magicicada septendecula*, little 17-yr cicada	Current study	…	6,521	518.9	C: 96.8% (S: 93.4%, D: 3.4%), F: 2.4%, M: 0.8%	22,785
Hemiptera	Membracoidea	*Homalodisca vitripennis*, glassy-winged sharpshooter	Li et al. (2022)	GCA_021130785.2	2,305	168.8	C: 92.7% (S: 82.0%, D: 10.8%), F: 1.0%, M: 6.2%	22,591
Hemiptera	Cercopoidea	*Callitettix versicolor*, rice spittlebug	Chen et al. (2022)	GCA_022606455.1	975	5.6	C: 94.7% (S: 92.3%, D: 2.3%), F: 0.5%, M: 4.7%	21,937
Hemiptera	Membracoidea	*Nephotettix cincticep*s, rice green leafhopper	Yan et al. (2021)	GCA_023375725.1	753	85.4	C: 97.0% (S: 95.2%, D: 1.8%), F: 2.0%, M: 1.0%	14,337
Hemiptera	Aphidoidea	*Acyrthosiphon pisum*, pea aphid	Li et al. (2019)	GCA_005508785.2	542	132.5	C: 93.3% (S: 89.7%, D: 3.6%), F: 1.9%, M: 4.8%	20,307
Hemiptera	Gerroidea	*Gerris lacustris*, common water strider	Wellcome Sanger Institute (2023)	GCA_951217055.1	938	76.4	…	…

Assembly BUSCO results = C, complete; S, single copy; D, duplicates; F, fragmented; M, missing.

## Supplementary Material

Supplementary material is available at *Genome Biology and Evolution* online.

## Supplementary Material

evae001_Supplementary_Data

## Data Availability

The final assembly (filtered and scaffolded), all corresponding annotation files, and the plot of telomeric regions can be found in a Figshare repository (doi:10.6084/m9.figshare.24488050). The final assembly is also currently being processed on NCBI under BioProject PRJNA966940. The raw reads, original unscaffolded assembly, Hi-C data and unfiltered assembly are available publicly on DNAZoo's website (https://www.dnazoo.org/assemblies/magicicada_septendecula).
