# Supplementary material for: Chromosome-Level Genome Assembly and Annotation of a Periodical Cicada Species: Magicicada septendecula
Source: Genome Biol Evol. 2024 Jan 8;16(1):evae001. doi: 10.1093/gbe/evae001 (PMC10799293; doi:10.1093/gbe/evae001)
Supplement: evae001_Supplementary_Data [file evae001_supplementary_data.pdf]

TABLE S1: Chromosome Breakdown

**Chromosome**	**Length (bp)**
1	1,456,256,088
2	926,935,479
3	615,102,008
4	525,418,745
5	500,509,132
6	493,883,959
7	489,529,673
8	483,657,605
9	444,160,652
X	368,691,587

TABLE S2: Paired-end, short-read RNA-Seq data provided to EASEL annotation pipeline.

Accession	Species	Total Reads (Before QC)	Total Reads (After QC)	Mapping Rate	Instrument	Size (Mb)
DRR055529	*Magicicada tredecim*	22,086,780	22,078,654	81.29%	Illumina HiSeq 4000	868.2
DRR055516	*Magicicada cassinii*	21,666,500	21,654,974	87.45%	Illumina HiSeq 4000	856.9
DRR055519	*Magicicada neotredecim*	22,101,494	22,092,126	82.28%	Illumina HiSeq 4000	869.3
DRR055520	*Magicicada septendecula*	21,924,338	21,912,260	94.83%	Illumina HiSeq 4000	2200
DRR055514	*Magicicada septendecim*	85,009,400	85,000,848	81.50%	Illumina HiSeq 2000	5900

TABLE S3: EnTAP Functional Annotation Statistics

Total Sequences: 8362163813

Similarity Search (RefSeq: complete.protein.faa.208.dmnd, NR: nr_protein.206.dmnd)

Total unique sequences with an alignment: 5726046701

Total unique sequences without an alignment: 2636117112

Gene Families (eggnog4.1)

Total unique sequences with family assignment: 8167358447

Total unique sequences without family assignment: 19485366

Total unique sequences with at least one GO term: 6808547306

Total unique sequences with at least one pathway (KEGG) assignment: 2125913776

Totals

Total unique sequences annotated (similarity search alignments only): 305744

Total unique sequences annotated (gene family assignment only): 2443812490

Total unique sequences annotated (gene family and/or similarity search): 8197859191

Total unique sequences unannotated (gene family and/or similarity search): 16434622

TABLE S4: Structural annotation quality assessment of EASEL

	Unfiltered	Filtered	Filtered (Longest Isoform)
Number of genes	140729	22785	22785
Number of transcripts	303929	83621	22785
Number of single exon genes	94901	3796	3934
mean exons per transcript	3.7	8.6	5.9
mean gene length	59856	173996	131586
Mono:multi ratio	2.07	0.200	0.209
BUSCO completeness (insecta)	99.7% [S:20.8%,D:78.9%]	96.4% [S:24.9%,D:71.5%]	91.2% [S:89.2%,D:2.0%]
70/70 reciprocal BLAST (RefSeq)	-	68.5%	59.4%

Table S5: Specimen collection details

Knox Co. TN

35º 57’ 22.72” N, 83º 51’ 58.08” 727’ elevation

IJAMS Nature Center trails just to the east of the Nature Center, near Tennessee River

23 May 2021, C. Simon, S. Chiswell

1 M. septendecula male, 2 M. septendecula females

Wilkes Co. NC

36º 11’ 8” N, 81º 4’ 51” W, 1020 ft. elevation

on River Road just north of the Yadkin River south of NC 268 (Elkin Highway)

24 May 2021, C. Simon, S. Chiswell

3 M. septendecula males.

Table S6

Link for the full interactive Hi-C contact map:

https://tinyurl.com/27woqjas

Figure S1: Blobplot

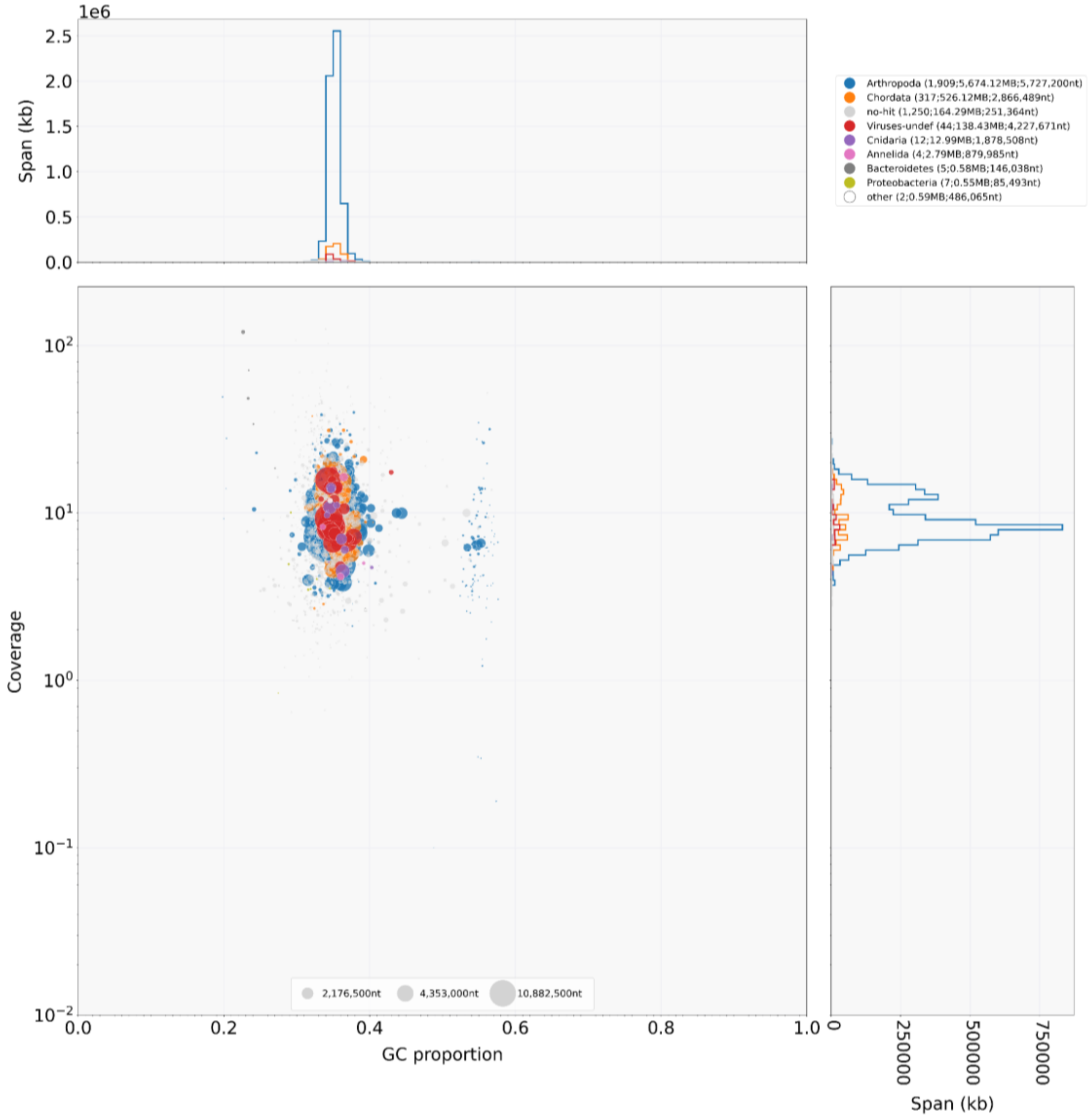

Figure S2: Telomeric-like regions

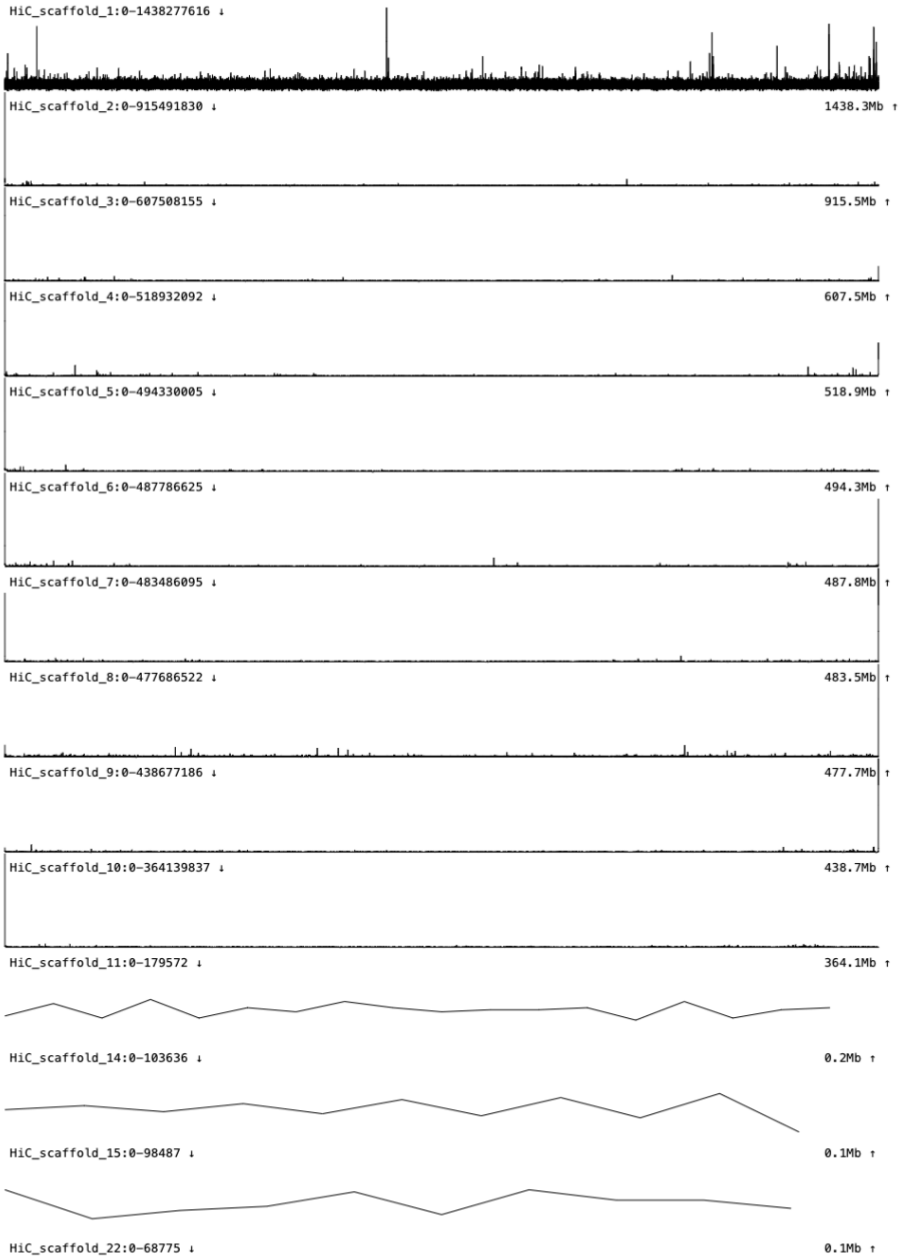

Sharp peaks found on both ends of the first 10 scaffolds represent high likelihood of telomeric regions. In the remaining shorter scaffolds (11,14, 15, 22), the consistent lines without peaks on the ends indicate no telomeres found. The full plot generated by tidk can be found in SVG format in the figshare repository (10.6084/m9.figshare.24488050).
